# Supplementary material for: Facile 3D Boron Nitride Integrated Electrospun Nanofibrous Membranes for Purging Organic Pollutants
Source: Nanomaterials (Basel). 2019 Sep 27;9(10):1383. doi: 10.3390/nano9101383 (PMC6836025; doi:10.3390/nano9101383)
Supplement: Supplementary file 1 [file nanomaterials-09-01383-s001.pdf]

Supporting Information

Facile 3D Boron Nitride Integrated Electrospun Nanofibrous Membranes for Purging Organic Pollutants

Dai-Hua Jiang [1,2,3,†], Pei-Chi Chiu [1,†], Chia-Jung Cho [1], Loganathan Veeramuthu [1], Shih-Huang Tung [2], Toshifumi Satoh [3], Wei-Hung Chiang [4], Xingke Cai [5] and Chi-Ching Kuo [1,*]

[1] Institute of Organic and Polymeric Materials, Research and Development Center of Smart Textile Technology, National Taipei University of Technology, 10608 Taipei, Taiwan; d06549013@ntu.edu.tw (D.-H.J.); chie92714@gmail.com (P.-C.C.); ppaul288@yahoo.com.tw (C.-J.C.); anloga947715@gmail.com (L.V.)

[2] Institute of Polymer Science and Engineering, National Taiwan University, 106 Taipei, Taiwan; shtung@ntu.edu.tw (S.-H.T.)

[3] Faculty of Engineering, Hokkaido University, Sapporo 060-8628, Japan; satoh@eng.hokudai.ac.jp (T.S.)

[4] Department of Chemical Engineering, National Taiwan University of Science and Technology, 10607 Taipei, Taiwan; whchiang@mail.ntust.edu.tw (W.-H.C.)

[5] Institute for Advanced Study, Shenzhen University, Shenzhen Guangdong 518060, China; cai.xingke@szu.edu.cn (X.C.)

[†] These authors contributed equally to this work

Correspondence: kuocc@mail.ntut.edu.tw; Tel: 886-2-27712171 ext. 2407

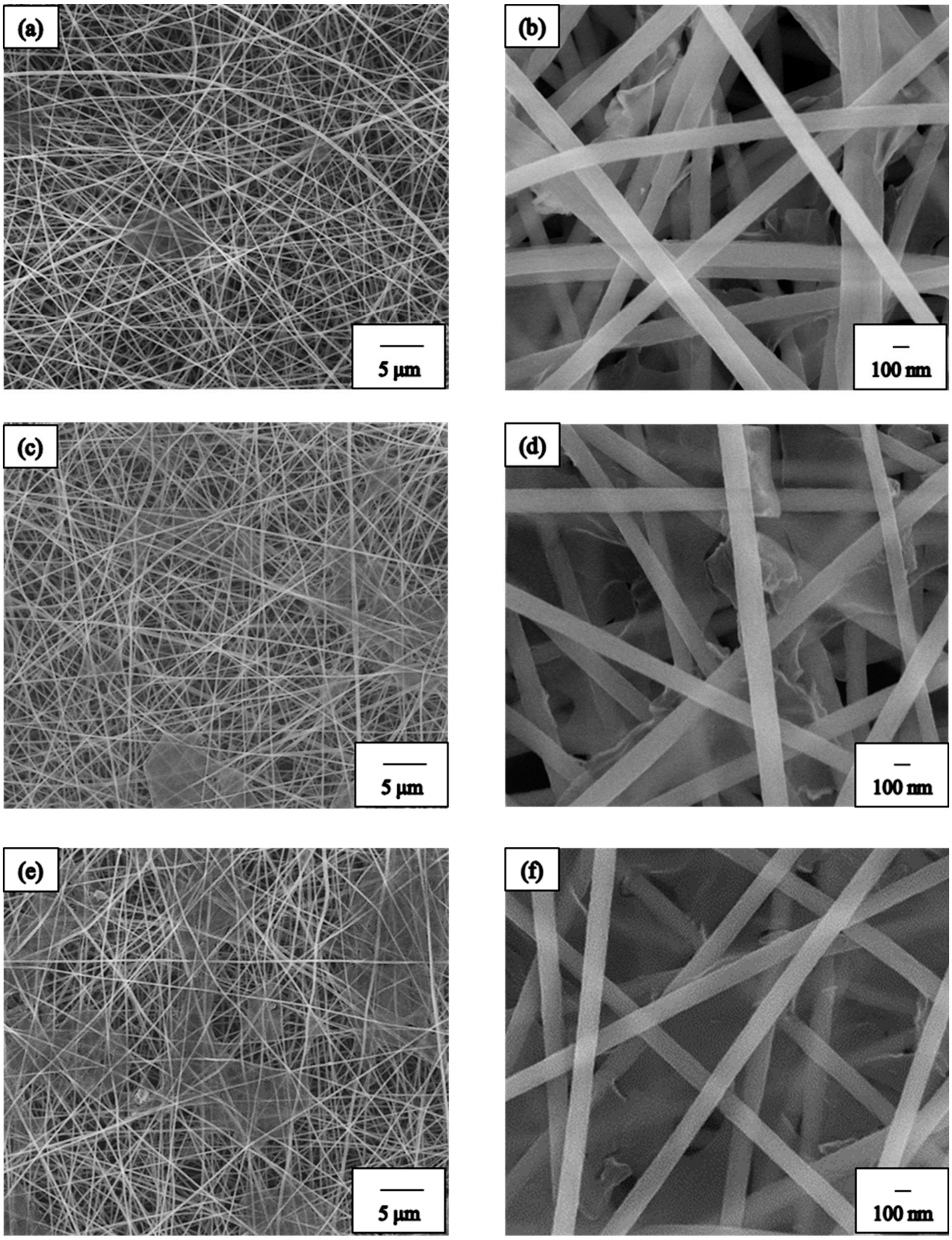

Figure S1. FE-SEM images of BN/PAN ES NFs with different spray coating time, the BN ES nanofiber membrane with the spray coating time (a, b) 10 s, (c, d) 30 s, (e, f) 60 s, is observed by the low and high magnification.

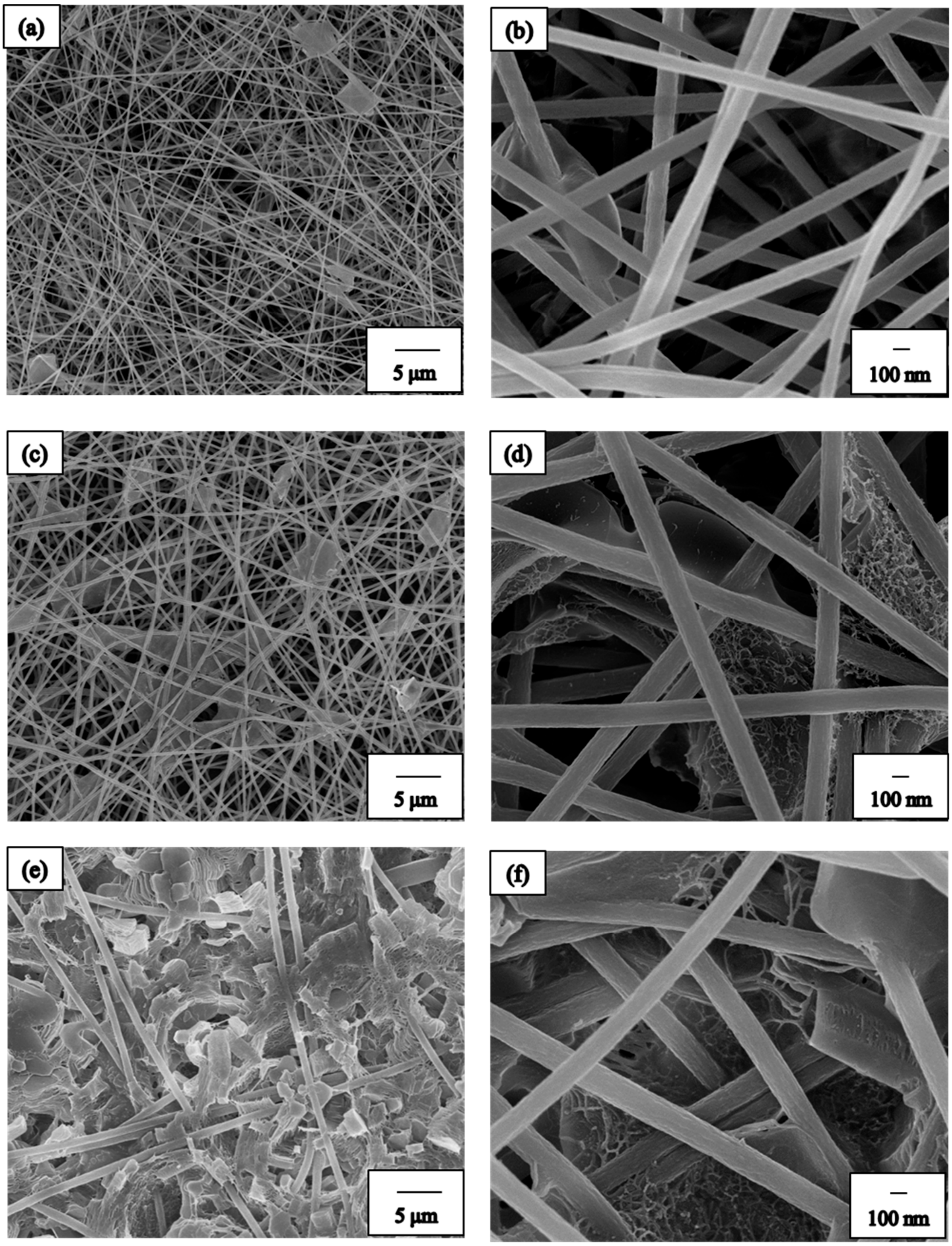

Figure S2. FE-SEM images of BN structure about the different ratio of the BN precursor, and the B_2O_3 and CH_4N_2O were respectively mixed with molar ratio (a, b) 1:10, (c, d) 1:5, (e, f) 1:1 in the low and high magnification,

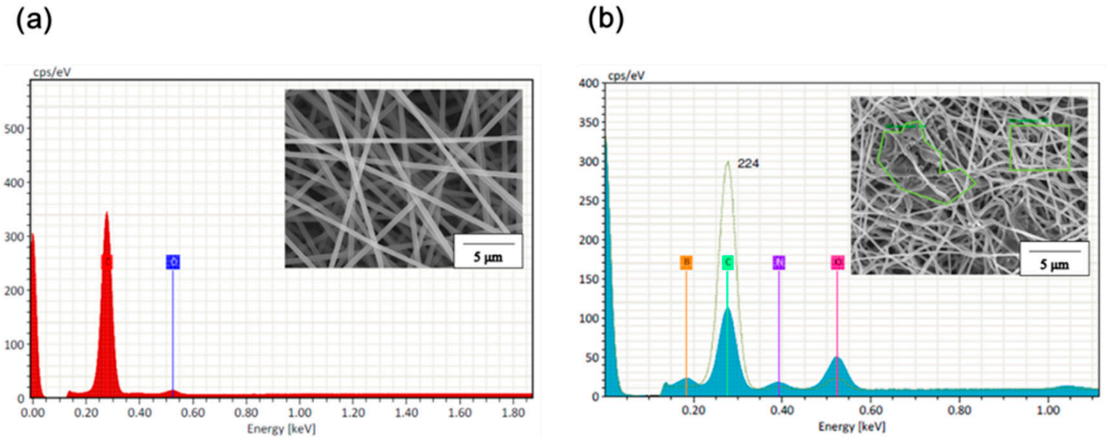

Figure S3. The EDX of (a) pure PAN carbon NFs and (b) BN-PAN composite ES NFs.

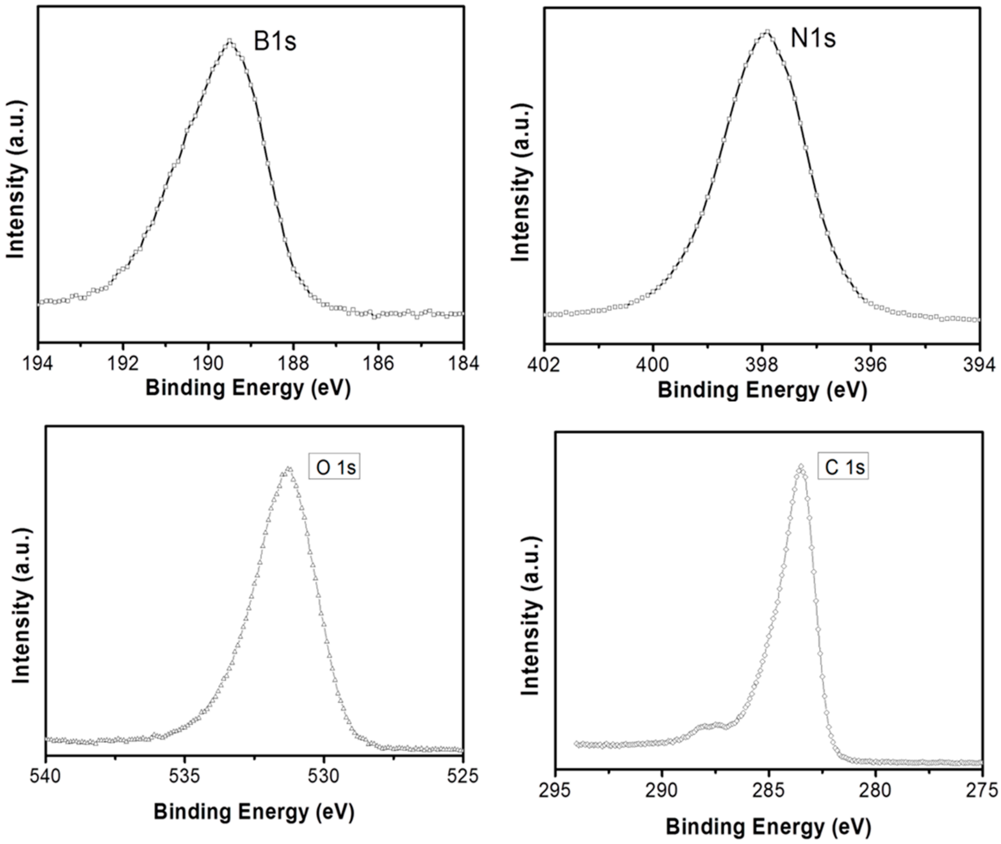

Figure S4. The X-ray photoelectron spectra (XPS) of BN-PAN composite ES NFs.

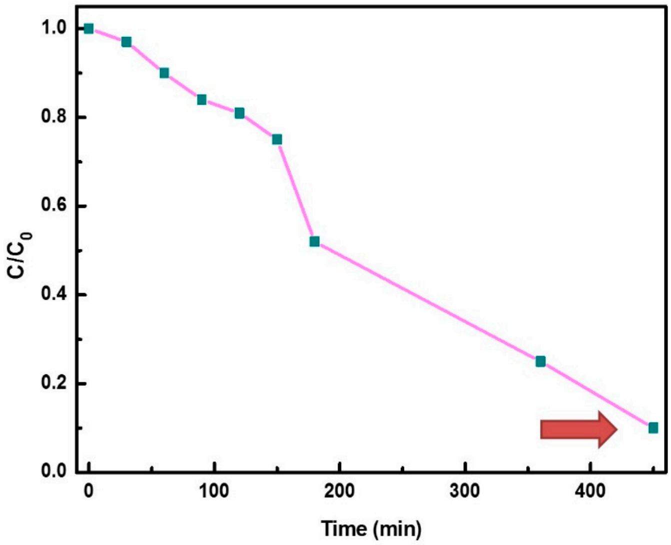

Figure S5. The removal efficiency of the 3D BN NF membrane in Rh B aqueous solutions after 450 mins.

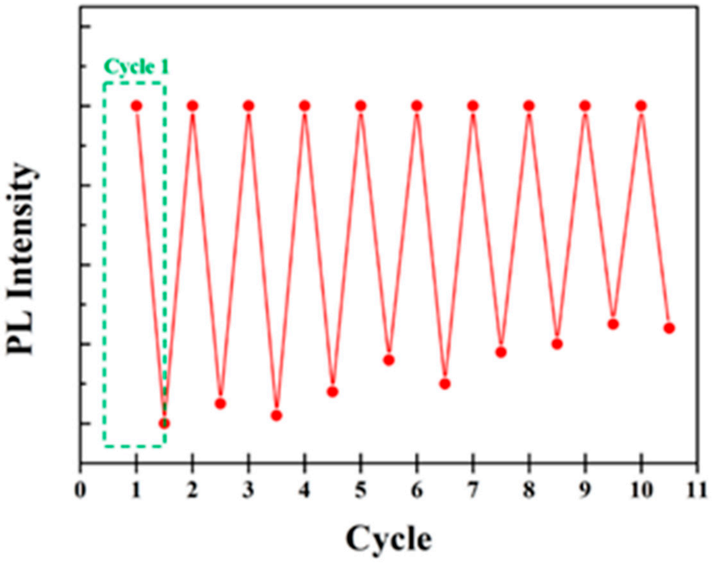

Figure S6. The membrane can be recycled and reused for at least 10 times (the plot of the PL intensity vs time for the 10 cycles).
